# Element substitution by living organisms: the case of manganese in mollusc shell aragonite

**DOI:** 10.1038/srep22514

**Published:** 2016-03-09

**Authors:** Analia L. Soldati, Dorrit E. Jacob, Pieter Glatzel, Janine C. Swarbrick, Jochen Geck

**Affiliations:** 1 Institut für Geowissenschaften, Johannes Gutenberg-Universität, Becherweg 21, D-55099 Mainz, Germany; 2 Department of Earth and Planetary Sciences, Macquarie University, North Ryde NSW 2009, Australia; 3 European Synchrotron Radiation Facility, F-38043 Grenoble Cedex 9, France; 4 Chemistry and Physics of Materials, Paris Lodron University Salzburg, Hellbrunner Str. 34, 5020 Salzburg, Austria

## Abstract

Determining the manganese concentration in shells of freshwater bivalves provides a unique way to obtain information about climate and environmental changes during time-intervals that pre-date instrumental data records. This approach, however, relies on a thorough understanding of how manganese is incorporated into the shell material –a point that remained controversial so far. Here we clarify this issue, using state-of-the-art X-ray absorption and X-ray emission spectroscopy in combination with band structure calculations. We verify that in the shells of all studied species manganese is incorporated as high-spin Mn^2+^, i.e. manganese always has the same valence as calcium. More importantly, the unique chemical sensitivity of valence-to-core X-ray emission enables us to show that manganese is always coordinated by a CO_3_-octahedron. This, firstly, provides firm experimental evidence for manganese being primarily located in the inorganic carbonate. Secondly, it indicates that the structure of the aragonitic host is locally altered such that manganese attains an octahedral, calcitic coordination. This modification at the atomic level enables the bivalve to accommodate many orders of magnitude more manganese in its aragonitic shell than found in any non-biogenic aragonite. This outstanding feature is most likely facilitated through the non-classical crystallization pathway of bivalve shells.

Trace elements in the skeletal hard parts of aquatic animals are sensitive recorders of environmental parameters, thus enabling the reconstruction of environments and climates of the past. Since the development of the earliest paleo-thermometers, which provided high-resolution proxy records from Sr/Ca ratios in aragonitic coral skeletons^1^, the number of trace element proxies multiplied, now comprising elements such as Mg, Cd, U, Zn, B, Ba and Mn. Paleoenvironmental parameters accessible with these tools are not restricted to temperature, but include changes in seawater pH (B^2^), freshwater input and ocean upwelling (Ba^3^,^4^), as well as the paleonutrient distribution in the oceans (Cd, Zn^5^). Manganese, in particular, can be used as a proxy for freshwater runoff^6^ and phytoplankton blooms, because of its close link to photosynthesis^7^,^8^. Determining Mn/Ca ratios in bivalve shells, allows the reconstruction of hydrologic-climatic regimes in lakes that pre-date instrumental records^7^.

An underlying assumption when using these proxy records for quantitative environmental reconstructions is that trace elements are accommodated in the carbonate phase via processes governed by equilibrium thermodynamics^9^ or kinetically controlled inorganic partitioning^10^. Interestingly, aragonitic shells of freshwater bivalves can contain Mn-concentrations well above 1000 μg/g^7^,^11^. This is a puzzling observation and strongly contrasts with the extremely limited uptake of Mn into non-biogenic aragonite^12^,^13^. At the same time it is well-known that manganese can readily replace calcium in non-biogenic calcite up to a point where all the Ca is exchanged and isostructural MnCO_3_ (rhodochrosite) is realized. This dichotomy between aragonite and calcite has previously been associated to the small ionic radius of Mn, which is better accommodated in calcite than in the aragonite host structure^14^. However, apart from these steric effects, the coordination chemistry of Mn has to be considered as well^15^: the atomic Mn 3d valence states are strongly anisotropic and aligned with respect to three orthogonal directions. In calcite, the O-ligands are situated exactly along these directions (cf. Fig. 1d), which leads to large orbital overlaps and strong covalent Mn-O bonds. This is not the case in aragonite. Here the O-ligands are not situated along the three orthogonal directions (cf. Fig. 1b) and therefore overlaps and the covalent bonding are significantly weaker. The energy gain due to covalent bonding in aragonite is therefore smaller than in calcite or, in other words, Mn is chemically more stable in the octahedral environment of calcite.

Major deviations between the non-biogenic materials and their biogenic counterparts are not restricted to Mn in bivalve shells, but comprise a large range of isotopic and elemental rations across many animal species^13^. None of them are currently understood. The underlying reason for the observed discrepancies between biogenic and non-biogenic carbonate may be found in their different formation mechanisms: Biogenic calcite and aragonite (cf. Fig. 1) that make up the skeletal hard parts are organic-inorganic nano-composites, frequently formed under extensive physiological control on the shape and composition of the minerals^16^. Up to now, the mechanistic details of their formation are still poorly understood. Growing evidence, however, indicates that many biogenic carbonates form via transient metastable precursor phases, such as amorphous calcium carbonate (ACC^17^,^18^,^19^). This stepwise crystallization pathway presents an energetically advantageous alternative to direct crystallization from solution, because of the low activation barriers between transient intermediate phases^20^. Although yet virtually unexplored, this pathway potentially influences the uptake and fractionation of key elements into the bio-carbonate. High Mg-calcite, for example, with up to 40 Mol% MgCO_3_ found in the calcitic teeth of echinoids^21^ can only be produced via ACC *in vitro*, but not through classical crystallization pathways^22^,^23^.

Furthermore, while there is strong evidence for the formation of mollusc shells from ACC^24^, the role of Mn as well as its speciation in the mature biomineral is still enigmatic^25^. Manganese is a particularly important element as it carries out critical functions as a catalyst in proteins and an activator in enzymes^26^ and thus could be partly accommodated in the organic macromolecules present in the bio-carbonate. The Mn-speciation in aragonitic shells has therefore been discussed controversially, and different authors proposed Mn to be associated with the organic moiety in the shell^25^,^27^ or its speciation in the aragonite lattice either as Mn(II)^28^,^29^ or as Mn (III)^30^.

In order to clarify these issues and to work toward an explanation towards the unusually high Mn concentrations in biogenic aragonite, we performed state-of-the-art K-edge X-ray absorption spectroscopy (XAS) and X-ray emission spectroscopy (XES) to determine the coordination and chemical bonding of Mn in different aragonitic freshwater bivalve shells.

## Results

### X-ray absorption spectroscopy

XAS data at the Mn K-edge were recorded for 10 different bivalve shells (Table 1 and Fig. 2). In this type of measurement the X-ray absorption cross-section is measured as a function of the incoming photon energy (hν_1_). As can be observed in Fig. 2a, all data sets exhibit the same characteristic features, including a pre-edge peak at 6.540 keV, a strong absorption peak at 6.552 keV (the so-called white line) and further common features at higher energies.

The pre-edge peak arises from transitions from the Mn 1s level into unoccupied orbitals that have mainly Mn 3d character (cf. Fig. 2e). These transitions are therefore very sensitive to the configuration of the 3d valence shell of the Mn-absorber. The identical intensity and shape of the pre-peak therefore already indicate that the configuration of the 3d-shell of Mn is the same for all bivalve samples studied here. This agrees well with our earlier results for *Diplodon chilensis patagonicus*^31^.

The 1s→4p transitions cause the main absorption edge above 6.550 keV. Since the 1s core-electron is excited into the 4p-bands, the main edge is not directly related to the configuration of the Mn 3d-valence shell. Instead, the 4p-states are rather delocalized rendering them sensitive to the local environment of the Mn-sites. In this way, the XAS main edge becomes sensitive to the local structure of the host around the Mn site.

The data in Fig. 2a shows that all major XAS features are very similar for all studied shells, although there are slight differences, especially in the intensity of the white line. We attribute the latter to different degrees of local disorder in the various samples^18^. Nonetheless, the similarity of the 1s → 4p XAS clearly indicates that the environment of Mn is very similar in all bivalve shell samples.

**Figure 1:**
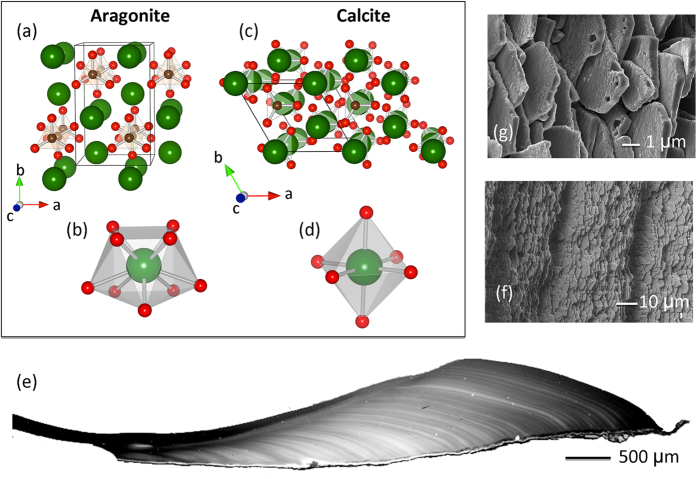
Schematic crystal structure of the CaCO_3_ polymorphs aragonite and calcite. Aragonite (**a**) has a crystal structure with orthorhombic symmetry (space group Pnma)^47^ where the Ca-site is nine-fold coordinated by O with site symmetry m, as shown in (**b**). In contrast, the crystal structure of calcite (**c**) is trigonal, space group R-3c^48^. In this case, calcium is octahedrally coordinated by O with site symmetry 32 (**d**). (**e**) Polished section of a freshwater bivalve shell of *Diplodon chilensis patagonicus* showing the seasonal banding with light bands formed in winters and dark bands formed in summers. (**f**) Secondary electron image of nacre layers indented by annual growth cessations that form during the annual reproductive period of the organism. (**g**) Secondary electron image of a broken surface of mother-of-pearl in the shell showing the typical nacre platelets.

At this point it is very interesting to compare the bivalve XAS to that of Mn within geological carbonate hosts. Interestingly, all geological aragonites that were studied in this work exhibited Mn-concentrations well below the detection limit of the present XAS experiment (ca. 10 μgg^−1^). It is also known that it is very difficult, if not impossible, to synthesize Mn-substituted aragonite^12^, because the crystal chemistry prohibits the incorporation of Mn into aragonite. The calcistructure, on the other hand allows for the presence of high Mn contents, as Mn is much less stable in the nine-fold coordination of aragonite (Fig. 1b) than in the octahedral coordination of calcite (Fig. 1d) for reasons outlined above.

**Figure 2:**
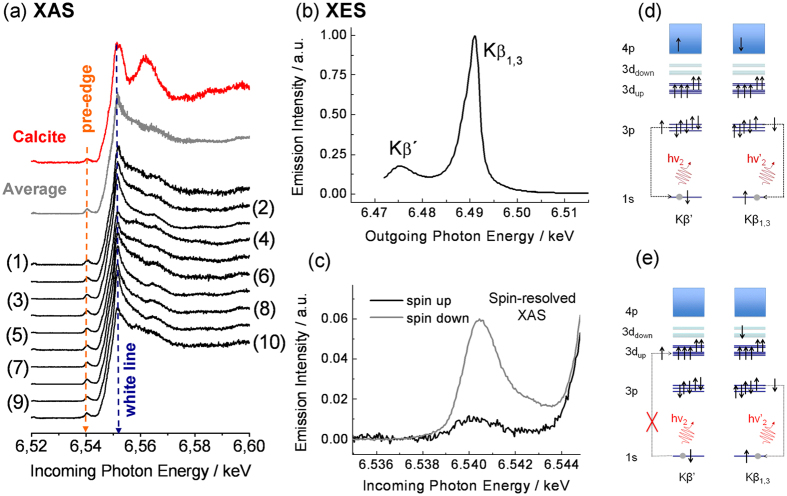
X-ray absorption, Kβ X-ray emission and spin-resolved X-ray absorption measurements. (**a**) XAS data for the studied bivalve samples. See Table 1 for the full names and description of the samples. The data 20 eV below and 60 eV above the white line has been used to normalize the different data sets. Calcite absorption is shown in red. The 10 shell spectra were averaged (grey curve) to allow better comparison with the reference material. (**b**) Kβ’ and Kβ_1,3_ main emission lines of the bivalve samples. The splitting of these two lines can clearly be observed and the total Kβ-lineshape agrees well with Mn^2+^ in a high-spin configuration. (**c**) Average shell spectrum of the spin-resolved XAS across the pre-edge peak. The spin-up absorption is strongly suppressed revealing a dominant 3d^5^ high-spin configuration of Mn. (**d**) Sketch of the decay processes that lead to the Kβ’ and Kβ_1,3_ main lines. Note that the total spin of the 3p-shell is different in both cases, resulting in a large exchange splitting of the lines. (**e**) Illustration of the spin-resolved XAS. Note that the spin-up 1s hole cannot be created (left), which is the necessary for the Kβ’ decay (cf. panel **d**). A spin-down hole can be created, enabling the Kβ_1,3_ decay illustrated on the right.

As can be seen in Fig. 2a, the pre-edge feature of the shells is identical in shape and intensity to that of the Mn-doped calcite, providing evidence that the 3d-configuration is the same in both types of samples. In contrast to this, the main edge at higher incoming photon energies is very different, in agreement with the established fact that the studied bivalve shells are not composed of calcite, but the mineral part consists of aragonite.

### Main Kβ-emission and spin-resolved XAS

**Figure 3:**
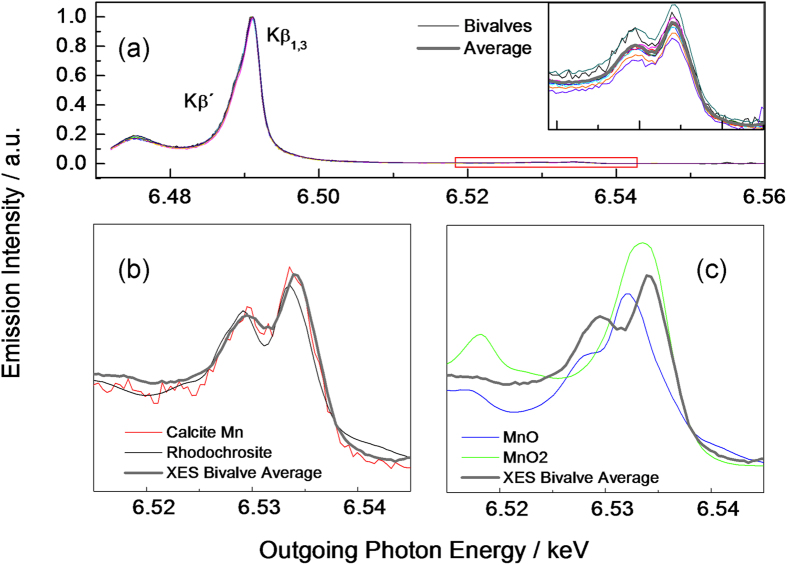
Valence-to-core X-ray emission. (**a**) Extended view of the bivalve Kβ X-ray emission, including the weak valence emission satellite at high emission energies (inset). Comparison of the bivalve data (averaged) to different Mn-bearing carbonate (**b**) and oxide (**c**) reference samples. The lineshape of the bivalve average spectrum is identical to that of Mn-doped calcite and rhodochrosite.

In order to obtain more information about the configuration of the 3d-valence shell of Mn, we performed XES and spin-resolved XAS. The emission process causing the Kβ main lines is illustrated in Fig 2d. After the 1s core hole has been created via photoionization, the resulting highly excited state can decay via a 3p→1s transition, i.e. the 1s core hole is filled by a 3p-electron. There are two types of final states that can be reached in this way: one where the unpaired spin of the 3p-shell, S_3p_, is parallel to the total 3d spin, S_3d_, and one where the two spins are antiparallel. Due to the presence of a strong exchange interaction between the 3p and 3d electrons, the final state energy is different for S_3p_ and S_3d_ being parallel and antiparallel. As a result, the two decay channels sketched in Fig. 2d correspond to different hν_2_ of the emitted photon. This is the origin of the well-known splitting of the main Kβ-emission lines, which is shown for our samples in Figs. 2b and 3a. For historical reasons the low-energy and high-energy features are referred to as Kβ’ and Kβ_1,3_, respectively, as indicated in Figs. 2b and 3a.

The lineshape and the splitting of the Kβ-main lines depend strongly on the valence and the spin-state^32^. As demonstrated in Fig. 3a the Mn Kβ-main lines were all found to be identical within the experimental error, further supporting that the electronic state of Mn is the same in all the biominerals examined in this study. Furthermore, the lineshape found for the bivalve materials agrees very well with that of Mn^2+^ in a 3d^5^ high-spin state^33^. This is the first direct evidence that Mn is incorporated in the aragonite biominerals as Mn^2+^.

To verify this conclusion we also performed spin-resolved XAS. By measuring photons with hν_2_ corresponding to the Kβ’ emission line, decays into the final states with S_3p_ and S_3d_ antiparallel are measured, whereas by setting hν_2_ to the Kβ_1,3_ emission line selects mainly final states with parallel S_3p_ and S_3d_^34^. The photo absorption preceding the Kβ-decay conserves the total spin (no spins are flipped), which has important consequences: the Kβ’-final state can only be reached if a spin-up 1s electron is excited by the incoming photon. Likewise, the Kβ_1,3_-final state is reached after a spin-down 1s electron absorbed the incoming photon. In this sense, one can perform spin-resolved XAS by choosing the appropriate hν_2_.

As illustrated in Fig. 2e, the pre-edge absorption of a spin-up 1s-electron is not possible for a 3d^5^ high-spin state and, consequently, Kβ’ emission is strongly suppressed. Spin-down 1s-electrons, however, can be excited, allowing for strong Kβ_1,3_ emission. This is a unique property of the 3d^5^ high spin state, which enables its identification via spin-resolved XAS.

The corresponding data for the studied bivalve shells is presented in Fig. 2c, where the spin-resolved XAS is shown as a function of the incoming photon energy hν_1_ across the absorption pre-peak. It can clearly be observed that the spin-up absorption is strongly suppressed as compared to the spin-down absorption, unambiguously identifying a dominating 3d^5^ high-spin configuration of the Mn-substituent.

### Valence-to-core emission spectroscopy

More detailed information about the chemical environment and bonding of Mn within the aragonite host can be obtained from valence-to-core XES, which arises from the decay of a valence electron with p-character to the Mn 1s core hole.

The technique therefore does not probe the transition metal d-orbitals but the occupied p-density of electronic states just below the Fermi level, which is mainly formed by electrons from ligand atoms. Since the valence states have a lower binding energy than the transition metal 3p-levels, the energy hν_2_ of the emitted photons is larger as compared to those emerging from the main Kβ-decay channels described above. In addition to this, the cross section for valence-to-core emission is smaller than for the main Kβ-decay. The valence emission thus results in weak high-energy satellites of the stronger main emission lines (Fig. 3a). The sensitivity of valence-to-core XES for studying the ligand environment has been demonstrated by various authors for various systems^35^,^36^. Also in the present case, the valence-to-core emission is very sensitive to the chemical environment of Mn, as can clearly be observed by comparing MnO to MnO_2_ (Fig. 3c).

As demonstrated in the inset of Fig. 3a, we could observe valence satellites for all measured shell samples. These data show that the valence-to-core line shapes of all studied shell materials coincide within the experimental error, implying that the chemical environment of Mn, i.e., the coordination symmetry and the type of ligand, in all these samples is the same. In addition to this, the comparison presented in Fig. 3b further reveals that the valence-to-core line shapes of the bivalve materials are identical to the calcite and rhodochrosite reference samples within the experimental error. This yields the major outcome of the present study: manganese in the aragonitic biominerals formed by freshwater bivalves is, in all studied cases, locally coordinated by an octahedron of six O-atoms belonging to 6 different CO_3_ molecules. The global aragonite structure of the biominerals (where the Ca^2+^ is surrounded by 9 nearest-neighbor O-atoms) is therefore altered locally around the Mn-sites in such a way that an octahedral coordination as in calcite is established (Fig. 1).

### Density functional theory

We performed model calculations by means of density functional theory (DFT) to obtain a microscopic interpretation of the valence-to-core XES line shape and to further support the sensitivity of this method (details are provided in the methods section). We first focus on the density of states (DOS) calculated for rhodochrosite and Mn-substituted aragonite. This quantity characterizes the electronic band structure in terms of the number of electronic states in a given energy region.

**Figure 4:**
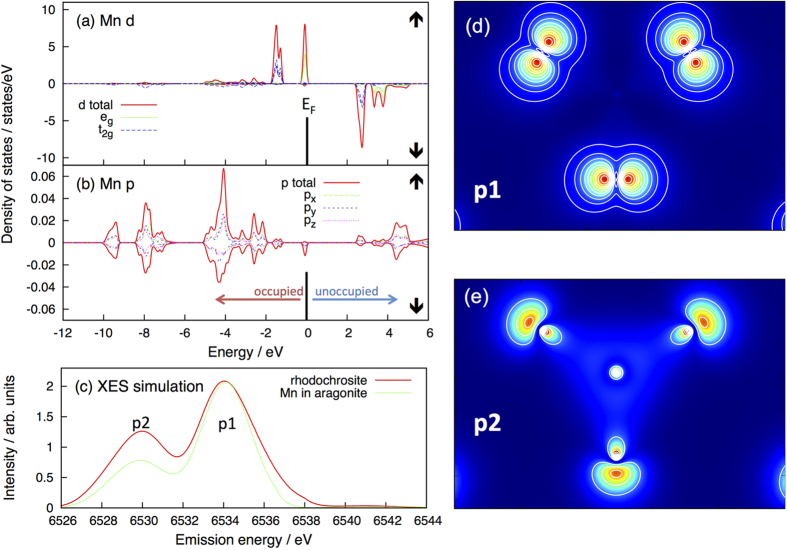
Density functional theory calculations. (**a,b**) Density of states for the spin-up Mn-site of rhodochrosite, projected on d- (**a**) and p-symmetry (**b**). (**c**) Simulated valence-to-core emission for rhodochrosite and Mn doped aragonite. (**d**,**e**): spatial distribution of the charge density corresponding to the p-bands p1 and p2, plotted in the plane of the CO_3_ molecule (blue: low, red: high). Most of the charge density is located on the outer oxygen ligands, which coordinate the C-site in the centre. It can be seen that p2 has a stronger C-O bonding than p1 in agreement with the higher binding energy of p2.

For example, the calculated DOS of the Mn 3d-states (d-DOS) displayed in Fig. 4a shows at which binding energies the Mn 3d-states occur in the DFT band structure. As expected on symmetry grounds for an octahedral coordination^37^, the 3d-levels of Mn are split into threefold degenerate t_2g_- and two-fold degenerate e_g_-states. Noting that the Fermi level E_F_ separates occupied from unoccupied states, it can be observed that all spin-up states (upper panel) are occupied, whereas all spin-down states (lower panel) are unoccupied. Our DFT calculations therefore yield a high spin-polarization of Mn in accordance with the strong Hund’s rule coupling. In fact, the calculated d-DOS corresponds to a 3d^5^ high-spin configuration of Mn. This is in excellent agreement with the experimental results described in the previous paragraphs.

In addition to the 3d-levels, there are also 2 main bands with p-character associated with Mn. This is demonstrated in Fig. 4b, where the DOS for the electronic states with p-symmetry is shown (p-DOS). The first band, p1, is located between about −2 eV to −5 eV binding energy, while the second, p2, lies between −7 eV and −12 eV. These two bands correspond to two different molecular orbitals of the CO_3_ complex. The calculated charge density distributions of p1 and p2 within the plane of the CO_3_ are shown in Figs. 4d,e. The fact that these states also appear in the p-DOS of Mn implies that these molecular orbitals hybridize significantly with Mn.

Turning to the simulation of the valence-to-core XES it is important to note that our DFT calculations provide a realistic description of the electronic ground state, while the photoionization process involved in XES creates an excited state. The simulation of XES based on such DFT calculations therefore neglects effects caused by the core-hole and, in particular, the screening of the core-hole is not taken into account. While this has important quantitative consequences, it does not play a role for the qualitative arguments given below. At this level we can therefore take the valence-to-core emission intensity to be proportional to the occupied p-DOS of Mn, because the corresponding p-electrons fill the core-hole via dipole transitions. This is a common approach that has been also used in earlier studies^38^.

Accordingly, we broadened the occupied Mn p-DOS by 1 eV in order to simulate the experimental width of the lines and shifted the resulting curve on the energy axis as to match the experimental emission energies. The results of this calculation are shown in Fig. 4c. As can be seen in this figure, the two bands in the p-DOS described above result in two peaks in the valence emission spectra. The first peak around 6535 eV can therefore be attributed to transitions of electrons in orbital p1 into the 1s core-hole, whereas the second peak around 6530 eV is caused by the corresponding transitions of electrons occupying orbital p2 (cf. Figs. 4d,e). Both the calculated relative intensity as well as the splitting of these peaks is in very good agreement with the experimental result.

The above results demonstrate that the valence-to-core XES line shape is directly determined by the hybridization of Mn with the ligands. Changing the geometry of the ligand coordination is therefore expected to change the valence emission line shape significantly. In order to verify this, we also performed a calculation for Mn in an aragonite host. As shown in Fig. 4c, this change in geometry has a significant effect in the valence-to-core emission. Most notably the relative intensity of the two peaks originating from p1 and p2 changes considerably. The present model calculation therefore demonstrates that this experimental method allows discriminating different local coordination geometries around the transition metal for one and the same type of ligand.

The fact that the measured valence-to-core emission line shapes of all the bivalve shells agree almost perfectly with that of the rhodochrosite and Mn-substituted calcite reference sample (Fig. 3b) therefore unambiguously reveals that Mn in the biominerals is coordinated by an CO_3_-octahedron. A different coordination, as the one in an aragonite environment, would result in significant deviations from the calcite reference.

## Discussion

To be able to use the environmental information provided by proxy elements to their full potential it is critical to understand the mechanisms behind the uptake and storage in the biomineral. This study provides new microscopic insights, thereby pinpointing important processes that govern the Mn-uptake in the shell materials. Specifically, we show that different freshwater aragonitic bivalve shells exhibit essentially identical XAS and XES spectra, which provides direct proof that all of them bear Mn in the same crystal environment, independent of bivalve type, provenance, or if recent or archaeological.

We unambiguously identified a dominant Mn^2+^ high-spin state of manganese in all the bivalve shell materials studied here. Most importantly, our valence-to-core emission spectroscopy data reveal that Mn^2+^ is coordinated by an octahedron of CO_3_-complexes, very similar to the local environment of Mn substituted into calcite. Note that this method is not only sensitive to the symmetry of the local Mn-environment, but also to the type of ligand. Our results hence imply that, although the global structure of the shell material corresponds to aragonite, the local structure around Mn is altered in such a way as to obtain an octahedral coordination by the O-ligands. This conclusion is supported by the fact that the covalent bonding of a high-spin Mn^2+^ is much stronger in the calcite environment as compared to the aragonite environment with much lower symmetry (cf. Fig. 1b,d). Due to this low symmetry, the Mn-O covalency is weak in non-biogenic aragonite, which explains the severely restricted Mn-uptake in these compounds. Obviously the latter poses a considerable challenge for organisms, which have to form bio-aragonite in aqueous solutions containing high concentrations of Mn.

This chemical restriction may be accommodated via a transient precursor crystallization pathway^17^,^18^,^39^,^40^, where the initially formed amorphous calcium carbonate phase is able to incorporate orders of magnitude more Mn than would be possible by non-biogenic aragonite. Upon crystallization of the aragonitic shell, the chemical stability of Mn in an octahedral coordination may drive the formation of calcite domains, which accommodate the Mn impurities in the aragonite host structure.

In contrast to direct ion-by-ion crystal growth in inorganic geological or common synthetic processes, the transient precursor pathway in bivalves therefore seems to enable the incorporation of large Mn-concentrations into the aragonite host lattices by changing the local symmetry around Mn at the atomic-scale. This is an excellent example of a so-called “vital effect”^41^ of physiology on mineralogy and chemistry.

## Methods

The bivalves used for this research (Table 1) were extant (i.e. not fossil) freshwater specimens of *Hyriopsis cumingii*, *Margaritifera margaritifera*, *Margaritifera falcata*, *Anodonta cygnea*, *Anodonta anatina* and *Unio tumidus*, as well as *Diplodon ch. patagonicus* from three different Patagonian lakes (Escondido, El Trébol and Morenito). One archaeological (sub-fossil) specimen of *Diplodon ch. patagonicus* from Lake El Trébol was investigated for comparison. Only the right valve of each specimen was used. Raman studies following the methodologies explained elsewhere^40^,^42^ were used to classify the different calcium carbonate polymorphs in the shells. All nacre or prismatic structures in these samples are composed entirely of aragonite. After cleaning with MilliQ water, the shells were air-dried and powdered shell nacre of the internal region of the ventral margin was extracted with a diamond-covered drill bit. After homogenization in an agate mortar the samples were mixed 1:1 with BN, and pressed into pellets of 5 mm diameter and approximately 1–2 mm thickness.

The reference compounds comprised synthetic substances and geological crystals (Table 1). CaCO_3_ of geological and biological origin is found commonly as the different crystal polymorphs calcite, aragonite, vaterite or amorphous calcium carbonate^39^. In this work we studied specifically the aragonite and calcite structures (Fig. 1). Non-biogenic aragonite shows always very low Mn concentrations (<10 μg/g), because uptake of Mn into the aragonite crystal structure is unfavourable^13^,^14^,^15^. Therefore also Mn-rich calcite and isostructural rhodochrosite (MnCO_3_) were included as Mn-bearing carbonate reference compounds.

The XAS and XES experiments were carried out at the beamline ID26 of the European Synchrotron Radiation Facility (ESRF) in Grenoble, France. Standard XAS measurements were performed by detecting the total fluorescence yield with an avalanche photo diode. For the spin-resolved XAS, the energy of the outgoing photons, hν_2_, was set to the maximum of the XES Kβ_1,3_ spectrum of the sample (for the spin down transition) and at the maximum of the Kβ´ line (for the spin up transitions). Further explanations regarding the spin-resolved XAS are given below. For the pre-edge region, the incoming photon energy, hν_1_, was scanned from 6.53 to 6.55 keV with an energy step of 0.05 eV. At least 3 scans were averaged for each sample. For the main edge and the extended absorption region, the hν_1_ was scanned between 6.52 and 6.60 keV, using an energy step of 0.1 eV.

The XES spectra were measured at an incident energy hν_1_=6.910 keV, i.e. well above the Mn K-edge. The Mn Kβ main XES spectra were acquired with hν_2_ between 6.472 and 6.505 keV and an energy step of 0.2 eV. Mn Kβ_2,5_ XES satellite lines were acquired with hν_2_ ranging from 6.503 to 6.550 keV in steps of 0.4 eV. At least five scans were averaged per sample. If not stated otherwise, XAS and XES spectra were normalized to an absorption intensity of 1 at the high-energy end of the XAS scans and to an emission area of 1 at the Mn-Kβ_1,3_ peak, respectively. The detection limit for Mn of the present setup was about 10 μg g^−1^.

### Model calculations

Spin-resolved density functional theory calculations were done employing the generalized gradient approximation for the exchange correlation potential^43^. No local onsite Coulomb interaction U was applied. The calculations were performed using the WIEN2K package^44^. We studied two different model systems: (i) rhombohedral rhodochrosite MnCO_3_ using the experimentally determined lattice and spin structure^45^ and (ii) the experimental lattice structure of aragonite^46^, where one of the four Ca-sites per unit cell has been replaced by Mn. In the latter model the spins of the Mn-sites in neighbouring unit cells were set to be parallel in order to reduce the computing time. This does not affect the qualitative features, which are of interest for the present study. For rhodochrosite and Mn-doped aragonite a mesh of 7 × 7 × 7 and 4 × 6 × 7 k-points was used in the irreducible part of the Brillouin zone. All calculations were well converged and, in the case of rhodochrosite, our results agree very well with previous band structure calculations^38^.

## Additional Information

**How to cite this article**: Soldati, A. L. *et al.* Element substitution by living organisms: the case of manganese in mollusc shell aragonite. *Sci. Rep.* **6**, 22514; doi: 10.1038/srep22514 (2016).

## Figures and Tables

**Table 1:** 

N º	Taxonomic Name	Origin	Collected	Pre-edge (keV)	Main edge (keV)	Oxidation state
1	*Anodonta anatina* (Unionoidea: Anodontinae)	University of Mainz	Recent	6540.5	6551.6	Mn (II)
2	*Anodonta cygnea* (Unionoidea:Anodontinae)	University of Mainz	Recent	6540.5	6551.6	Mn (II)
3	*Hyriopsis cumingii* (Unionoidea:Unionidae)	University of Mainz	Recent	6540.5	6551.6	Mn (II)
4	*Margaritifera falcata* (Unionoidea: Margaritiferidae)	University of Mainz	Recent	6540.4	6551.6	Mn (II)
5	*Margaritifera margaritifera* (Unionoidea:Margaritiferidae)	University of Mainz	Recent	6540.5	6551.7	Mn (II)
6	*Unio tumidus* (Unionoidea:Unionidae)	University of Mainz	Recent	6540.5	6551.6	Mn (II)
7	*Diplodon chilensis patagonicus* (Unionoidea: Hyriidae)	Lake Morenito	Recent	6540.4	6551.6	Mn (II)
8		Lake Escondido	Recent	6540.4	6551.6	Mn (II)
9		Lake El Trébol	Recent	6540.4	6551.6	Mn (II)
10		Lake El Trébol	Sub-fossil	6540.4	6551.6	Mn (II)
11	Calcite (natural)	University of Mainz	Mineral	6540.4	6551.6	Mn (II)
12	Rhodrochrosite (natural)	University of Mainz	Mineral	6540.2	6551.9	Mn (II)
13	MnO (synthetic)	Sigma-Aldrich	Compound	6540.2	6550.16553.8	Mn (II)
14	MnO_2_ (synthetic)	Sigma-Aldrich	Compound	6542.7	6553.86560.1	Mn (IV)

Details on samples (superfamily and family in brackets) and reference materials including peak positions. Valves 1-6 and the two mineral samples are from the collection of the University of Mainz. The *Diplodon chilensis patagonicus species* in lines 7-10 consist of three individuals live-collected in 2007-2010 and a sub-fossil shell from Lake El Trébol in 2002, provided by Dr. Adam Hajduk.
